# Exploratory study on factors influencing the introduction of complementary feeding amongst caregivers of children between 6 and 24 months of age in Polokwane, Limpopo province

**DOI:** 10.4102/safp.v65i1.5522

**Published:** 2023-02-20

**Authors:** Mabitsela H. Mphasha, Gerald Mokubela, Thendo Ramokotedi, Thapelo Kgari

**Affiliations:** 1 Department of Human Nutrition and Dietetics, Faculty of Healthcare Sciences, University of Limpopo, Polokwane, South Africa

**Keywords:** complementary feeding, factors, caregivers, children, social media

## Abstract

**Background:**

Complementary feeding should be introduced at six months to meet infants’ growing nutritional needs. Inappropriate complementary feeding poses threats to the health, development and survival of infants. The Convention on the Rights of the Child states that every child has the right to good nutrition. Caregivers should ensure that infants are fed properly. Factors such as knowledge, affordability and availability impact complementary feeding. Hence, this study explores factors influencing complementary feeding amongst caregivers of children between the age of six and 24 months in Polokwane, Limpopo province, South Africa.

**Methods:**

A qualitative phenomenological exploratory study design was used to collect data from 25 caregivers, using purposive sampling; the sampling size was dependent on data saturation. Data were collected through one-on-one interviews using voice recorders and field notes for non-verbal cues. Data were analysed using the eight steps of Tesch’s inductive, descriptive and open coding technique.

**Results:**

Participants had knowledge about when and what to introduce during complementary feeding. Participants alluded that availability and affordability, maternal beliefs about infant hunger cues, social media, attitudes, returning to work because of the end of maternity leave and painful breasts affect complementary feeding.

**Conclusion:**

Caregivers introduce early complementary feeding because of returning to work at the end of maternity leave and painful breasts. Additionally, factors such as knowledge about complementary feeding, availability and affordability, mother’s beliefs about child hunger cues, social media and attitudes influence complementary feeding.

**Contribution:**

There is a need to establish credible social media platforms to disseminate appropriate complementary feeding messages. The established credible social media platforms must be promoted, and caregivers must be referred from time to time.

## Background

At the age of 6 months, exclusive breastfeeding alone is no longer sufficient to meet infants’ growing nutritional needs.^1,2^ The transitional process from exclusive breastfeeding to family food, known as complementary feeding, should be introduced.^2^ Usually, complementary feeding covers a period between six and 24 months of the infant’s age with continuation of breast milk. However, breast milk can be continued beyond two years.^3^ This transition period is an extremely critical phase, especially in children’s growth and development. Studies have reported that adequate breastfeeding and optimal complementary feeding significantly contribute to promoting health and supporting growth whilst also enhancing brain development in these infants.^3,4^ Nutrient deficiencies and illnesses resulting from inadequate complementary feeding could contribute to increasing the global rates of undernutrition amongst children under five years of age.^5^ In 2020, 149 million children under the age of five years throughout the world were stunted, 45 million wasted and 38.9 million were overweight or obese.^6^ In South Africa, 27% of children are stunted and less likely to reach full growth and developmental potential.^7^ Undernutrition is particularly associated with 45% of child deaths in the world. Equally so, poor complementary feeding practices in the earlier phase of life may subsequently lead to detrimental health effects later in life. Studies often conclude that children who were poorly fed during the complementary feeding period are at a higher risk of obesity and non-communicable diseases.^5,6^

Infants under the age of 24 months need to be looked after and taken care of by mothers or caregivers. According to the World Health Organization,^3^ infants who are 6 months of age should start consuming safe and nutritionally adequate solids, semi-solids or soft foods whilst continuing to be breastfed until two years of age or beyond. As such, caregivers should ensure these by being responsive to the child’s cues for hunger and encouraging the child to eat. According to the United Nations ‘Convention on the Rights of the Child’, every child has the right to good nutrition.^8^ However, various studies in South Africa and other countries reported poor practices related to complementary feeding practices.^9,10^ Insufficient quantities and quality of complementary food, together with poor feeding practices, pose a threat to children’s health and nutrition.^9^

Complementary feeding could be influenced positively or negatively, depending on how it is done in line with the nutritional requirements. A study assessing factors affecting complementary feeding reported that caregivers introduced solids earlier because of the perception that they were not producing sufficient breast milk and that there was breast milk refusal by the infant, so they were encouraged by family members to do so.^11^ Furthermore, caregivers with more demands of household chores introduced complementary feeds sooner because they complained about having limited hours for breastfeeding.^11^

Factors such as maternal age, educational level and nutrition knowledge and identification of the signs that show the child’s readiness to explore new tastes and foods were found to contribute significantly to complementary feeding.^12^ In addition, the family socio-economic status also had impact on complementary feeding.^12^ Moreover, an Ugandan study indicated that mothers or caregivers who honoured the growth monitoring and promotion programmes for deworming and Diphtheria-Tetanus-Pertussis (DPT3) and measles vaccinations are at increased chances of feeding their children appropriately.^13^ This is attributed to their consistent interaction with healthcare practitioners who teach and support complementary feeding.^13^

A South African study conducted in Eastern Cape province cited affordability, household food availability and inadequate or lack of income as determining the food complementary feeding choices rather than their food preferences.^14^ The lack of knowledge amongst caregivers may lead to poor complementary feeding practices.^15^ Majority of caregivers stated that when infants cry a lot, having an increased appetite and wanting to be breastfed more often than usual are some of the signs that the child is ready for complementary feeding.^16^ So far, studies have not clearly and adequately explored factors influencing caregivers who are responsible to the feeding of children between the age of six and 12 months. Hence, the researchers conducted this study to explore factors influencing the introduction of complementary feeding amongst caregivers of children between six and 24 months of age in Polokwane, Limpopo province, South Africa’.

## Research methodology

### Research method and design

A qualitative method was adopted in conducting this study, using a hermeneutical phenomenological exploratory study design. The caregivers of children between the ages of six and 24 months were interviewed individually regarding their children’s complementary feeding practices and factors that influenced their complementary feeding were explored.

### Study period

This study was conducted over a period of two months, which includes data collection and analysis.

### Study setting

This study was conducted in Rethabile Clinic in Polokwane, Limpopo province. The clinic is situated in the urban area of the Polokwane Municipality, and close-by Polokwane Hospital. Families from various cultural background migrated from their rural areas to reside in Polokwane. The nurses indicated that over 50% of the children aged 6–24 months in their register are brought by guardians and that most introduce complementary feeding earlier. Therefore, the study setting was chosen because it can be easier to obtain participants meeting the inclusion criteria.

### Reflexivity

The researchers remained neutral during the process of data collection and used probing and reflexivity to get more data from participants by using statements such as ‘so what you are actually saying is…’

### Sampling and sampling size

In this study, the participants were or are caregivers of infants aged 6–24 months attending clinic for any service immunisation and charting weight for the under-five card in Rethabile Clinic. Recruitment and selection of participants took place at the clinic through verbal communication wherein face-to-face engagements occurred with potential participants at the clinic on the designated day for infant and young children services. Data collection were conducted within the two month period of the study. Twenty-five participants were purposively sampled. The sample size was dependent on data saturation. Data collection was discontinued following data saturation. The inclusion criteria for this study were as follows: caregivers must be over 18 years of age and speak English or Sepedi who attended the clinic for immunisation and growth monitoring of their children.

### Data collection instrument and procedure

Data were collected through one-on-one interviews using voice recorders. Interviews were conducted privately, and participants were alerted when voice recorders were switched on and off. The interviews were conducted in Sepedi because it is the dominant language in the area and interviewers were fluent in Sepedi. The data were gathered through unstructured interview guide with open-ended questions that were developed based on available literature. The grand tour question for the interview was ***‘****Kindly explain when and what made you introduce food to your children’.* In addition, the following questions were asked: ‘*Why did you introduce food at that age’ and ‘Why those food?’*. The data were collected by the researchers who engaged in community nutrition practicals at the clinic. Data collection bias that could have occurred during interviews with participants was minimised through probing and reflexivity. Based on the responses for each question, further probing and clarity-seeking questions were asked to obtain in-depth information. The probing questions were dependent on participants’ responses. Moreover, data from all participants were collected by two researchers from the Rethabile Clinic. Each interview lasted for about 15–30 min, and the whole data collection process was conducted within a period of one month.

### Pilot study

A pilot study was conducted involving four caregivers of children aged 6–24 months from a non-participating clinic before the main study was undertaken to pre-test the interview guide, and its findings did not inform any modification on data collection instrument. Data collected from participants used during the pilot study were not included in the main study because they were sampled from a non-participating clinic in Polokwane.

### Measures to ensure trustworthiness

Measures of rigour in this study were guaranteed through trustworthiness (see Table 1).

**TABLE 1:** Measures to ensure trustworthiness.

Criteria	Strategy	Applicability
Credibility	Prolonged engagement	The researcher interviewed respondents for about 15–30 min and probing questions were asked to obtain in-depth information. Researchers responsible for data collection were engaged on community nutrition practicals at Rethabile Clinic. Therefore, the researchers are well known in the area and stayed in the field for a month collecting data to build trust and rapport with participants.
	Triangulation	Researchers used both interviews and observations in collection of data.
	Member checks	It was done at the conclusion of each interview whereby the researcher summarised information and respondents validated the accuracy of the information.
Transferability	Data saturation	Data were collected until saturation was reached. Data saturation was reached with participant number 22 but an additional three were sampled to confirm saturation and thus the total number of respondents was 25. The data were collected for a period of 1 month, with interviews lasting between 15 min and 30 min each.
	Thick description	A detailed description of the study setting, participants and methodology were provided and shall permit other researchers to transfer findings.
Confirmability	Peer review	Data were collected by two researchers who independently analysed the data, agreed on themes and subthemes. Thereafter, a consensus meeting was convened with supervisors of the researcher and indent coder for confirmation of findings.
	Audit trail	A meeting was held with supervisors before and after data collection and analysis to guide the data collection process and agree on themes, respectively. Moreover, all the steps including materials, records and notes collected during the study will be kept safely for the next 5 years.
Dependability	Dense description of research methods	Full description of research methods and reporting of verbatim results make the findings dependable.
	Reliance on data collection tools and supervisors	The researchers relied on the voice recorder and field notes and also relied on supervisors.

### Data analysis

The recorded semi-structured interviews conducted in Sepedi were transcribed verbatim and translated to English, and the data were further presented to a language interpreter. The researcher began analysing the data after each data collection session. Additionally, after the whole data collection and analysis by the researcher, the data transcripts were submitted to supervisors. The researchers and supervisors met in a consensus meeting and conceded to themes and sub-themes. Respondents’ direct quotations are made and caught in italic format to support findings. Data were analysed using the eight steps of Tesch’s inductive, descriptive open coding technique,^17^ as shown in Table 2.

**TABLE 2:** Tesch’s eight steps of open coding.

Steps	Applicability
Step 1 – Reading through the data	The researchers carefully and repeatedly read transcripts of all participants and understood them and jotted down ideas.
Step 2 – Reduction of the collected data	The researchers scaled down the data collected to codes based on the existence or frequency of concepts used in the verbatim transcriptions.
Step 3 – Asking questions about the meaning of the collected data	The researcher asked himself questions about transcriptions of the interview, based on the codes that existed from the frequency of the concepts. Questions included ‘what is this about?’ and ‘what is the underlying meaning?’
Step 4 – Abbreviation of topics to codes	The researchers abbreviated topics that emerged as codes. These codes were written next to the appropriate segments of the transcription.
Step 5 – Development of themes and sub-themes	The researchers developed themes and sub-themes from coded data and associated texts and reduced the total list by grouping topics that relate to one another.
Step 6 – Compare the codes, topics and themes for duplication	The researchers reworked from the beginning to check the work for duplication and to refine codes, topics and themes where necessary.
Step 7 – Initial grouping of all themes and sub-themes	The data belonging to each theme were assembled in one column and a preliminary analysis was performed, which was followed by the meeting between the researchers and supervisors to reach consensus.
Step 8 – Recoding if necessary	A necessity to recode emerged as some of the themes reached independently were merged.

### Ethical considerations

Turfloop Research Ethical Committee (TREC) approved the study, with reference number: TREC/235/2021: undergraduate, and that Limpopo Department of Health granted permission to conduct the study. In addition, all participants provided written informed consent. Participation was voluntary. The participants were informed about their rights to withdraw from the study at any stage without penalty. Privacy and confidentiality of the participants’ data were also maintained.

## Results

### Demographic profile of the qualitative participants

Table 3 shows that 25 participants took part in the study, of which 24 were women and 22 were of the age between 19 and 35 years. Moreover, 13 caregivers were unemployed and that only 15 had tertiary education.

**TABLE 3:** Demographic profile of caregivers of children aged from 6 to 24 month.

Socio-demographic profile	Categories	*N* (25)	%
Age (years)	18–35	22	88
	> 36	03	12
Gender	Female	24	96
	Male	01	4
Employment status	Employed	12	48
	Unemployed	13	5
Education	High school education or less	10	40
	Tertiary education	15	60

Table 4 shows a theme regarding factors influencing complementary feeding, which had 6 sub-themes, namely, affordability, maternal beliefs about hunger cues, social media, end of maternity-leave and return to work, painful breasts, and attitudes towards introduction of complementary feeding at six months.

**TABLE 4:** Themes and sub-themes emerged from data analysis.

Theme	Subtheme
1. Factors influencing complementary feeding	1.1. Availability and affordability 1.2. Maternal beliefs about infant hungers cues 1.3. Social media 1.4. End of maternity leave and return to work 1.5. Painful breasts 1.6. Attitudes towards introduction of complementary feeding at 6 months

#### Theme 1: Factors influencing complementary feeding

Complementary feeding can be impacted negatively or positively by various factors, which subsequently determines the child’s health status and developmental progress. Participants in our study outlined factors that have influenced their practice of complementary feeding as evident in the following sub-themes that have emerged from this theme.

**Sub-theme 1.1: Affordability:** Affordability of food often determines food intake, including during complementary feeding. Consequently, adequate or inadequate food intake because of affordability and availability determines a child’s growth and development. Participants stated that their practice of complementary feeding is affected by affordability and availability as supported by the following quotes:

‘I can’t afford most of food such as infant cereals, so I don’t give different kinds of foods.’ (Participant 7, 36 years, male, unemployed)

‘[*T*]he mother of this child is late, and I only depend on the social grant and some financial assistance from relatives, I also have my own children who are still at school and depends on me. As such I cannot afford some of the healthy food this child needs.’ (Participant 24, 53 years, female, unemployed)

However, one participant stated that she affords and has no challenge of food unavailability, as supported by the following quote:

‘I don’t have problem with unavailability of food, I can afford it, food is always there for my child even when I go to work.’ (Participant 3, 24 years, female, employed)

**Sub-theme 1.2: Maternal beliefs about hunger cues:** Traditionally, there are cultural beliefs associated with children’s hunger cues, which mothers or caregivers influence through the introduction of complementary feeding. Participants of this study stated that when the baby cries a lot and grabs things into the mouth (mouthing), these are signs that the child is ready for the food, as supported by the following quotes:

‘I started my child with solid food at 3 months, because this child was crying nonstop, when I started giving food, she became better.’ (Participant 21, 28 years, female, unemployed)

‘[*M*]y mother-in-law told me to start giving solid food at 4 months, because the child was crying nonstop and used to grab everything and put in the mouth, so they said breastmilk was no longer enough.’ (Participant 15, 27 years, female, employed)

**Sub-theme 1.3: Social media:** Social media such as Facebook and Twitter are platforms where people discuss a wide range of topics including complementary feeding. The information on social media is easily accessible; however, the information on social media is dominantly people’s opinions and not scientifically proven. In spite of these, participants in this study alluded that they are reliant on social media on how to feed their children, as evident in the following quote:

‘I joined Facebook group about children where mothers discuss everything pertaining the child, that’s how I knew about starting complementary feeding before I even went to clinic.’ (Participant 3, 24 years, female, employed)

**Sub-theme 1.4: End of maternity leave and return to work:** Working women are usually afforded maternity leaves to breastfeeding and bond with their children. The period allocated to maternity leave differs, however, usually not equivalent to the 6 months period recommended for exclusive breastfeeding. Therefore, participants in this study stated that they had to initiate complementary feeding earlier, as supported by the following statement:

‘I started giving solid food at 4 months, because I had to go back to work.’ (Participant 25, 28 years, female, employed)

**Sub-theme 1.5: Painful breasts:** The discontinuation of breastfeeding can be influenced negatively by painful breasts, which will ultimately lead to the early introduction of complementary feeding. Participants in this study alluded that they introduced complementary feeding earlier to their children because of painful breasts, as supported by the following quote:

‘I started my child with complementary feeding early at 3 months, and because my breast were painful.’ (Participant 14, 28 years, female, unemployed)

**Sub-theme 1.6: Attitudes towards the introduction of complementary feeding at 6 months:** It is recommended that the appropriate age to introduce complementary feeding is at six months. Therefore, the attitudes of caregivers who are responsible for feeding children are important. In this study, participants showed positive attitudes towards the recommended age of introduction of complementary feeding and further indicated that they can encourage new mothers to introduce complementary feeding at six months. Below are some quotations by participants:

‘It is good to start complementary feeding at 6 months, since it helps children grow well. I will advise new mothers to introduce food to their children at six months.’ (Participant 2, 24 years, female, employed)

‘The period of exclusion breastfeeding/formula feeding can be challenging and tempting to introduce food earlier; however, I advise mothers to start solid food at 6 months with one food item at a time, it helps to know if their child is tolerating the food.’ (Participant 11, 22 years, female, employed)

## Discussion

Caregivers of children aged between six and 24 months are responsible for feeding of their children. Hence, it is of utmost importance to understand factors that impact the practice of complementary feeding, from the perspective of these caregivers. Therefore, the aim of this study was to explore factors influencing complementary feeding amongst caregivers of children between six and 24 months of age. Participants indicated that they started complementary feeding earlier because of painful breasts and return to work owing to the end of maternity leave, including maternal beliefs on hunger cues. Moreover, participants pointed out affordability and social media impact on the practice of complementary feeding. Attitudes of caregivers towards the introduction of complementary feeding at the age of 6 months were found to affect appropriate practices.

Participants in this study reported that they introduce complementary feeding earlier than the recommended 6 months.^3^ This was attributed to their beliefs on hunger cues, painful breasts and returning to work after completion of maternity leave, which does not correspond with the recommended appropriate age of introduction of complementary feeding. The findings of this study affirm previous studies conducted in various countries,^10,18^ including South Africa.^19^ The timing of introducing complementary feeding is crucial and constitutes the basis of appropriate feeding practices.^20^ Early or late introduction of complementary feeding is associated with undesirable short- and long-term health consequences later in life.^21^ Therefore, there is a need for primary healthcare facilities to establish infant and young child feeding support groups to improve the introduction of complementary feeding at an appropriate age. According to Rodríguez-Gallego et al.^20^ mother-to-mother support groups have been found to improve breastfeeding, leading to the introduction of complementary food at recommended six months. Nonetheless, complementary feeding can be affected by other factors; hence, it is critical for healthcare providers to extract other factors that may impact feeding during counselling. In addition, the reasons that led participants to introduce complementary feeding earlier demonstrate a need to strengthen education on complementary feeding or infant and young child feeding. At the same time, the findings highlight the need for the South African government to amend maternity to 6 months. Studies have demonstrated an association between prolonged maternity leave and improved exclusive breastfeeding.^22,23^

Social media was indicated in this study as one of the factors influencing complementary feeding. Social media platforms such as Facebook and Twitter have become ubiquitous, with more people easily accessing content through following links than direct searches.^24^ The use of social media is increasing significantly and reaches a large number of audiences. It was reported that social media also brings substantial changes in communication amongst organisations and individuals.^24^ Nonetheless, the use of social media for health promotion messages, including complementary feeding, has not been explored or harnessed fully. According to George et al.,^25^ social media has direct public health relevance because social networks could have an important influence on health behaviours and outcomes. According to George et al.,^26^ the World Health Organization recommends cost-effective interventions to solve health problems. Therefore, the Department of Health should establish credible social media pages dedicated to the promotion of appropriate complementary feeding. In addition, primary healthcare facilities should innovatively establish the social media platforms in local languages and share the links with all the caregivers within the records, as well as the public. These will minimise the communities or caregivers following links that are not based on scientific information. Moreover, these social media platforms must also have chat-box, wherein caregivers can ask clarities or questions. Therefore, there should be healthcare providers designated to manage social media pages or chat-boxes for prompt response to concerns or questions.

Participants in this study highlighted affordability as a contributing factor to the practice of complementary feeding. A study conducted in Nepal found that most caregivers attributed poor practice of complementary feeding to the unavailability of food because of unemployment.^27^ Social and economic factors are regarded as non-medical determinants of health.^28^ These factors could impact complementary feeding practices; therefore, there is a need to evaluate socio-economic factors of caregivers prior to developing an intervention to promote appropriate complementary feeding practices. It has been found that each parent or caregiver intends to feed their children appropriate and nutritious complementary food.^29^ However, affordability and availability, which are key drivers of food intake, prohibit implementation of such intentions, thereby becoming barriers to intake of nutritious foods.^30,31^ Worldwide, one quarter of the population are estimated that less than 1.5 billion persons cannot afford the cheapest possible nutrient-dense food,^32^ whilst 53% of the population in sub-Saharan Africa cannot afford to provide adequate and nutritious food to their children. Unaffordability of nutritious food, coupled with affordable non-nutritious foods, is found to be the main driver of poor quality of diet, contributing to increasing prevalence of malnutrition in children. From the age of six to 24 months, young children need nutrient-dense foods for their growth and brain development.^18,33^

Attitudes of mothers or caregivers have been recognised as the most important determinant of infant feeding practices.^34^ Participants in this study showed good attitudes towards complementary feeding and further indicated that they are willing to encourage new mothers to practise appropriate complementary feeding. On the contrary, other studies have reported poor attitudes amongst mothers negatively impacted on the practice of complementary feeding.^30,35^ It was also reported that improving attitudes of mothers through nutrition counselling and education can lead to improved infant and young child feeding practices and, consequently, improved child growth and development, more especially in settings recorded to have low maternal literacy.

## Conclusion

The following factors were found to influence the knowledge of caregivers of children between 6 and 24 months of age in Polokwane: (1) availability and affordability, (2) mother’s beliefs about child hunger cues, (3) social media and (4) attitudes towards the introduction of complementary feeding at six months. In addition, returning to work because of end of maternity leave and painful breasts was found to be a contributing factor for the early introduction of complementary feeding. There is a need to establish credible social media platforms in the local languages for the referral of caregivers within the records of primary health care facilities and the community. Moreover, the social media platforms should be equipped with healthcare providers designated to respond to questions in the chat-box promptly. The healthcare providers as primary implementers of infant and young child feeding should be skilled enough to extract factors or myth, during counselling, that could negatively affect complementary feeding.

### Recommendations

Establish credible social media platforms in local languages for referral of caregivers, and the need for having healthcare providers designated to clarifying questions in the social media chat-boxes.

### Limitations

The findings of this study cannot be generalised to represent the entire population of caregivers of children aged 6–24 months; however, it forms the basis for further research.
